# Uniparental disomy for chromosome 1 with *POMGNT1* splice-site variant causes muscle-eye-brain disease

**DOI:** 10.3389/fgene.2023.1170089

**Published:** 2023-06-05

**Authors:** Yi-Dan Liu, Dan-Dan Tan, Dan-Yu Song, Yan-Bin Fan, Xiao-Na Fu, Lin Ge, Wei Wei, Hui Xiong

**Affiliations:** ^1^Department of Pediatrics, Peking University First Hospital, Beijing, China; ^2^ Beijing Kangso Medical Inspection Co., Ltd., Beijing, China

**Keywords:** uniparental disomy (UPD), muscle-eye-brain disease (MEB), PomGnT1, dystroglycanopathy (DGP), splice-site variant

## Abstract

*POMGNT1*, encoding protein O-mannose beta-1,2-N-acetylglucosaminyltransferase 1, is one of the genes responsible for dystroglycanopathy (DGP), which includes multiple phenotypes such as muscle-eye-brain disease (MEB), congenital muscular dystrophy with intellectual disability, and limb-girdle muscular dystrophy Here, we report a case of MEB that is the result of a homozygous variant of *POMGNT1* that is revealed through uniparental disomy (UPD). An 8-month-old boy was admitted with mental and motor retardation, hypotonia, esotropia, early onset severe myopia, and structural brain abnormalities. A panel testing of genetic myopathy-related genes was used to identify a homozygous c.636C>T (p.Phe212Phe) variant in exon 7 of *POMGNT1* in the patient, a heterozygous c.636C>T variant in the father, and the wild type in the mother. Quantitative polymerase chain reaction (q-PCR) revealed no abnormal copy numbers in exon 7. Trio-based whole-exome sequencing (trio-WES) revealed a possible paternal UPD on chromosome 1 of the patient. Chromosomal microarray analysis (CMA) revealed a 120,451 kb loss of heterozygosity (LOH) on 1p36.33-p11.2, encompassing *POMGNT1*, and a 99,319 kb loss of heterozygosity on 1q21.2-q44, which indicated UPD. Moreover, RNA sequencing (RNA-seq) verified that the c.636C>T variant was a splice-site variant, leading to skipping of exon 7 (p.Asp179Valfs*23). In conclusion, to the best of our knowledge, we present the first case of MEB caused by UPD, providing valuable insights into the genetic mechanisms underlying this condition.

## Introduction

Dystroglycanopathy (DGP) is a group of autosomal recessive muscular dystrophies caused by O-glycosylation defects in alpha-dystroglycans. It has three phenotypes based on severity: type A (severe forms of congenital muscular dystrophy [CMD] with brain and eye abnormalities, including Walker-Warburg syndrome [WWS], muscle-eye-brain disease [MEB], and Fukuyama CMD [FCMD]), type B (CMD with or without intellectual disability), and type C (a mild form of limb-girdle muscular dystrophy [LGMD]) (Song et al., 2021).

MEB (OMIM 253280) was first described by Santavuori et al. (1977) in Finland in 1977. Typical clinical features include profound hypotonia at birth, motor and cognitive developmental delays, and ocular abnormalities. In 2001, the first gene responsible for MEB, *POMGNT1*, was identified by Yoshida et al. (2001) in Japan. In recent years, we have reported novel *POMGNT1* variants that cause MEB in Chinese patients and identified a novel copy number variation, g.6668-8257del of *POMGNT1*, as a founder mutation (Jiao et al., 2013; Fu et al., 2017). In our clinical and genetic analysis of a large cohort of Chinese patients with DGP, *POMGNT1* is the most common gene responsible for MEB (Song et al., 2021).

Homozygous variants or compound heterozygous variants lead to autosomal recessive inherited diseases, and disease-causing variants conventionally originate from both parents. However, in rare cases, the variant from one parent is the only cause, such as in uniparental disomy (UPD). UPD is defined as two copies of a whole or partial chromosome derived from one parent. These two copies can be maternal or paternal. Maternal UPD is approximately twice as common as paternal UPD (Benn, 2021). Here, we report a case of MEB due to a homozygous variant in *POMGNT1* revealed by paternal UPD.

## Case description

### Clinical data

An 8-month-old boy was admitted to the pediatric clinic of our hospital because of a “mental and motor developmental delay.”

He had a delay in motor development since birth and unsteadily raised his head at 4 months. He was unable to roll over, sit without support, or crawl at 8 months. He was unable to grip items on his own. He had delayed intellectual development. He could smile but could not babble. The parents had a non-consanguineous marriage, and no related disease was observed in the family history.

Physical examination revealed a head circumference of 43 cm, esotropia, hypotonia, and muscle weakness with limb muscle strength between level 3 and level 3+, predominantly proximal. The bilateral patellar tendon reflexes could not be induced. No pathological reflection of the Babinski’s sign was observed.

**FIGURE 1:**
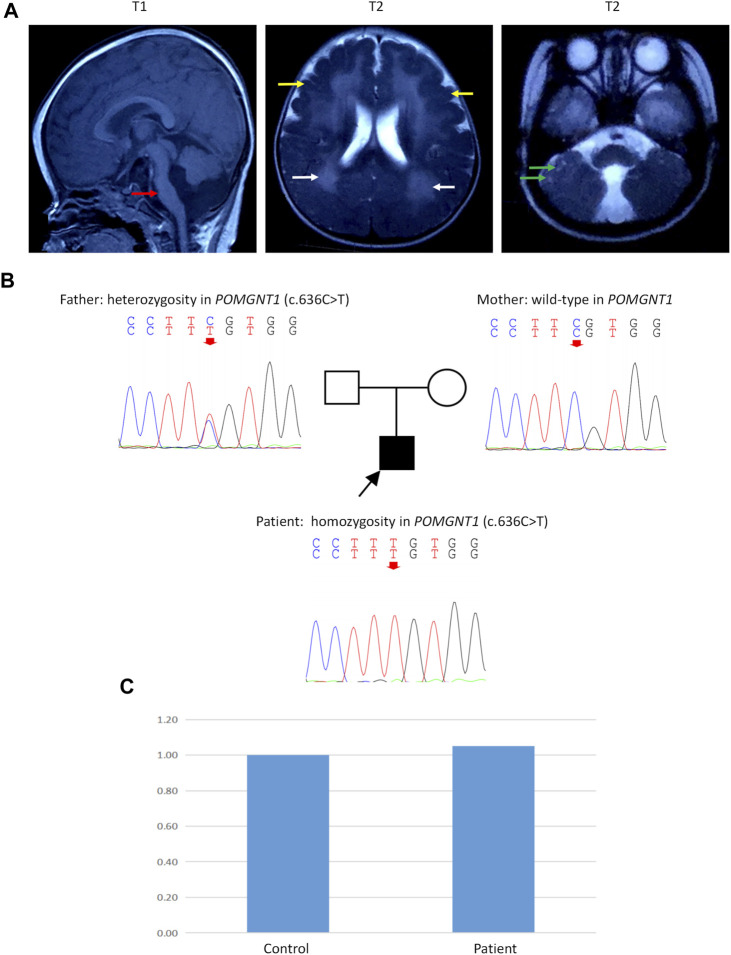
Brain magnetic resonance imaging (MRI), Sanger sequencing, and quantitative polymerase chain reaction (q-PCR). **(A)** Brain MRI of the patient at 8 months of age. T1: Cerebellar and brain stem hypoplasia with a characteristic flattening (red arrow). T2: Pachygyria-polymicrogyria of frontotemporal lobes (type “cobblestone pavement”) (yellow arrows) and white matter lesions (white arrows). T2: Multiple subcortical cerebellar cysts (green arrows). **(B)** Sanger sequencing showed a homozygous c.636C>T variant in exon 7 of the *POMGNT1* gene (NM_001243766) in the patient. His father had a heterozygous c.636C>T variant, and his mother had no variant in this site. **(C)** Q-PCR of DNA showed a normal copy number in exon 7 of *POMGNT1* in the patient.

Serum creatine kinase (CK) levels were elevated (1799 IU/L). Brain magnetic resonance imaging (MRI) showed cerebellar and brain stem hypoplasia with a characteristic flattening, pachygyria-polymicrogyria of frontotemporal lobes (type “cobblestone pavement”), white matter lesions, and multiple subcortical cerebellar cysts (Figure 1A), which were typical brain changes in DGP (Borisovna et al., 2019). Examination of blood amino acids (aa) and urine organic acids revealed no significant abnormalities. Optic examination revealed extreme myopia.

He achieved rolling over, sitting without support, and standing with support at approximately 1 year of age. He was able to crawl at the age of 2. When he was 3 years old, he could walk several steps with support. After 3 years of age, there was no significant improvement in motor development. At the last follow-up, the boy was 6 years old and was unable to walk independently. He could say “baba” and “mama” but no other words. He wore corrective glasses for extreme myopia (the uncorrected visual acuity was between 0.01 and 0.05).

### Genetic findings

Peripheral blood samples were collected from the patient and his parents after obtaining informed consent. A panel testing of genetic myopathy-related genes was first performed, and the candidate variants were verified using Sanger sequencing. The gene panel testing identified a homozygous c.636C>T (p.Phe212Phe) variant in exon 7 of the *POMGNT1* gene (NM_001243766) in the patient, and the pathogenicity of this variant has been reported previously (Bouchet et al., 2007). Sanger sequencing revealed that the father had a heterozygous c.636C>T variant, but the mother had no variant at this site (Figure 1B).

**FIGURE 2:**
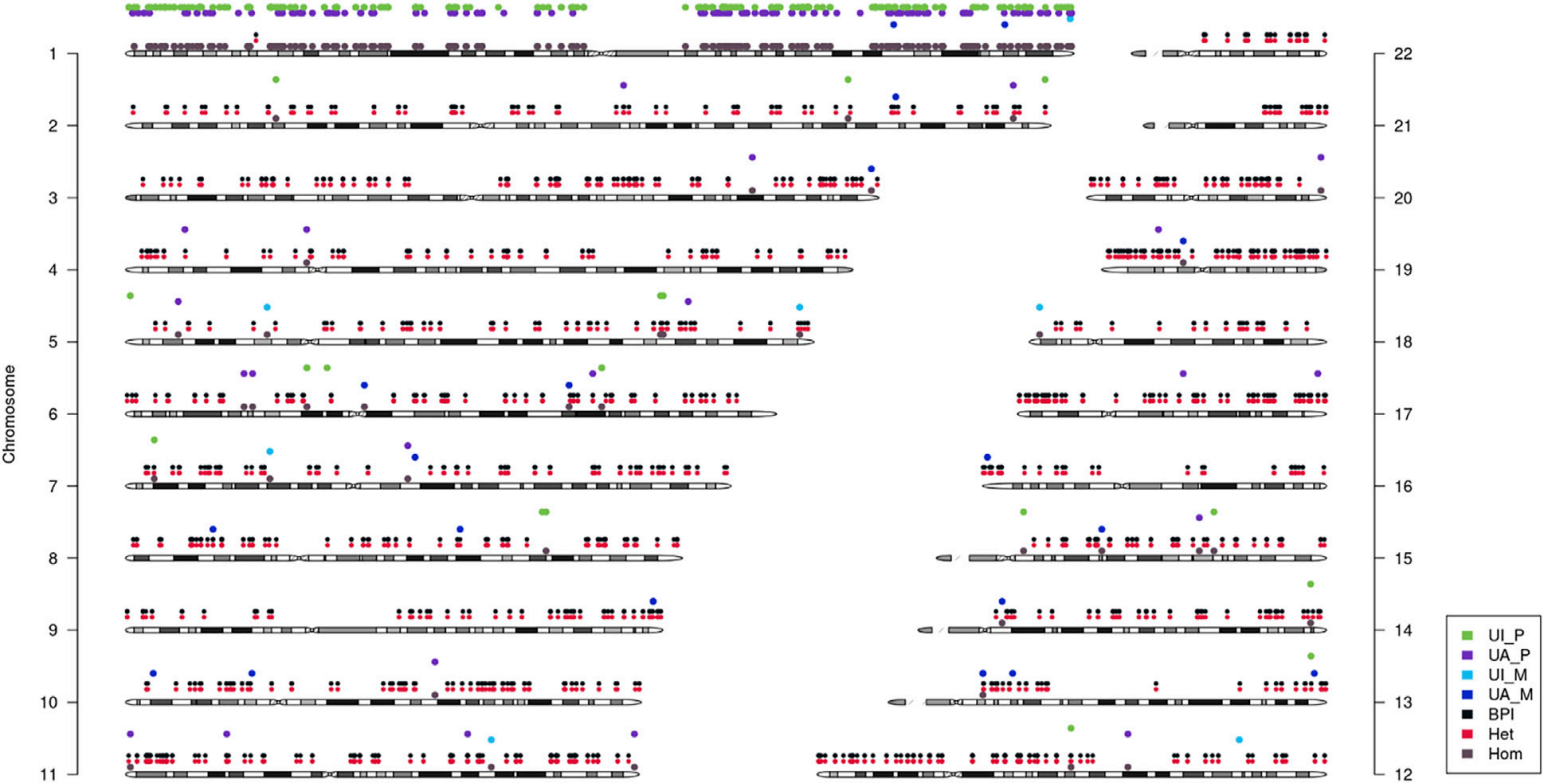
Analysis of uniparental disomy (UPD) using trio-based whole-exome sequencing (trio-WES). It showed that paternal UPD existed on chromosome 1. UI_P: Uniparental Paternal Isodisomy. UA_P: Uniparental Paternal Heterdisomy. UI_M: Uniparental Maternal Isodisomy. UA_M: Uniparental Maternal Heterdisomy. BPI: Bi-Parental Inheritance. Het: Heterozygous. Hom: Homozygous.

**FIGURE 3:**
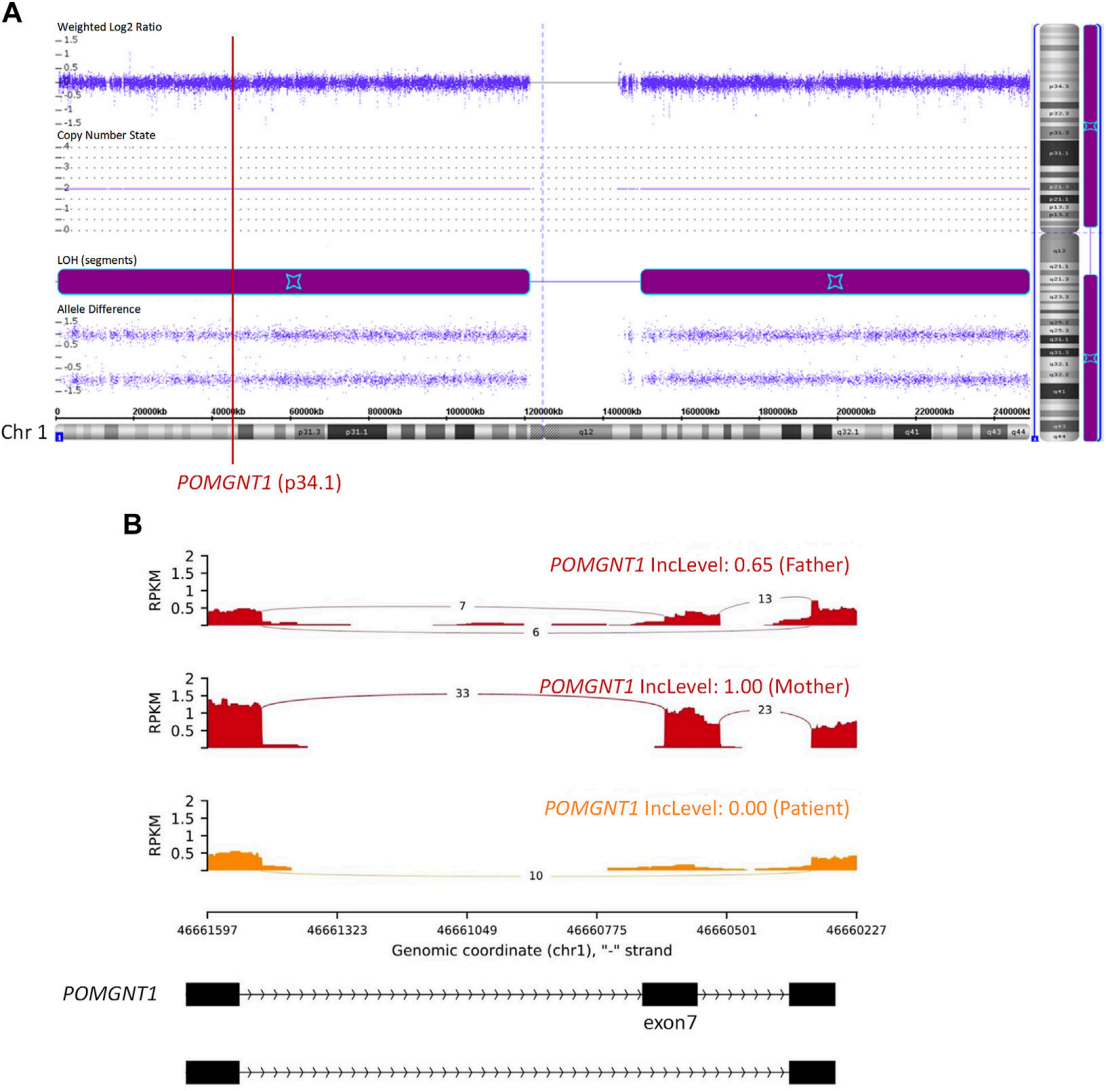
Chromosomal microarray analysis (CMA) and RNA sequencing (RNA-seq). **(A)** CMA showed a 120,451 kb loss of heterozygosity (LOH) on 1p36.33-p11.2 (888,658_121,339,317) and a 99,319 kb LOH on 1q21.2-q44 (149,879,544_249,198,164). The former encompassed the *POMGNT1* gene. **(B)** RNA-seq showed skipped exon 7 in the *POMGNT1* mRNA of the patient and the father. RPKM: Reads Per Kilobase per Million mapped reads. IncLevel: Inclusion Level.

Then, quantitative polymerase chain reaction (q-PCR) of DNA was used to identify whether there was an abnormal copy number in exon 7 of *POMGNT1* in the patient. However, no abnormalities were found (Figure 1C), suggesting that there may be another cause of the homozygous variant. Next, trio-based whole-exome sequencing (trio-WES) was performed to identify other variants and UPD. As a result, a possible paternal UPD on chromosome 1 of the patient was identified using trio-WES data analysis (Figure 2), which revealed the homozygous variant, and no other pathogenic variants were detected. Finally, chromosomal microarray analysis (CMA) of the patient revealed a 120,451 kb loss of heterozygosity (LOH) on 1p36.33-p11.2 (888,658_121,339,317), encompassing *POMGNT1*, and a 99,319 kb LOH on 1q21.2-q44 (149,879,544_249,198,164) (Figure 3A). Thus, UPD was ascertained.

The c.636C>T (p.Phe212Phe) variant is synonymous. RNA sequencing (RNA-seq) of peripheral blood samples was performed to determine whether splicing was affected. It was confirmed to cause the skipping of exon 7 in the *POMGNT1* mRNA (Figure 3B), resulting in a frameshift change (p.Asp179Valfs*23). Based on the ACMG guidelines (Richards et al., 2015), c.636C>T is considered a pathogenic variant.

## Discussion

MEB is a phenotype of DGP, which is characterized by hypotonia at birth, brain structural abnormalities, and ocular malformations (Jiao et al., 2013). Considering the typical symptoms in the muscles, eyes, and brain of the patient, making a diagnosis of MEB is clear.

*POMGNT1* is the most common gene responsible for MEB (Song et al., 2021). A homozygous c.636C>T (p.Phe212Phe) variant in exon 7 of *POMGNT1* was found in the patient. However, only his father had a heterozygous c.636C>T variant and his mother was the wild type. Initially, copy number variation (CNV), a *de novo* variant, or UPD were considered. Then, the result of q-PCR excluded CNV of exon 7 on DNA level. Finally, paternal UPD on chromosome 1 was confirmed by using trio-WES and CMA. RNA-seq revealed skipped exon 7 in the patient and the father on mRNA level. RNA-seq also showed a lower RPKM (Reads Per Kilobase per Million mapped reads) value of the father and the patient than the mother. For the three samples of this family were sequenced at the same time and the sequencing depth was standardized in calculation, the lower RPKM value of the father and the patient may be caused by nonsense-mediated mRNA decay.

The POMGNT1 protein contains 660 aa and has four domains: an N-terminal cytoplasmic tail (aa 1–37), a transmembrane domain (aa 38–58), a stem domain (aa 59–300), and a catalytic domain (aa 301–660) (Akasaka-Manya et al., 2004). The c.636C>T (p.Asp179Valfs*23) variant found in the present study leads to the loss of part of the stem domain and all of the catalytic domain. In previous studies, the c.636C>T variant was reported in fetal forms of type II lissencephaly, a severe clinical presentation of CMD and in an 8-year-old girl presenting CMD with clinical features compatible with MEB or severe FCMD (Bouchet et al., 2007; Oliveira et al., 2008). Different from the patient in the present study, the 8-year-old girl with a homozygous c.636C>T variant in the literature had epilepsy in addition to hypotonia, visual impairment, and brain structure abnormalities (Oliveira et al., 2008).

UPD occurs with an estimated overall prevalence of 1 in 2,000 births (Nakka et al., 2019), not all of which causes phenotypic consequences. UPD can lead to clinical presentation when it involves disrupting imprinting (chromosomes 6, 7, 11, 14, 15, and 20) and uncovering recessive alleles in blocks of isodisomy (Nakka et al., 2019; Benn, 2021). UPD for any chromosome is associated with an increased risk for a recessive disorder since it can result in an affected child when only one parent is a carrier of a pathogenic variant (Del Gaudio et al., 2020). LOH is one mechanism that leads to UPD. Somatic cell recombination between chromatids results in two populations of cells with reciprocal segments with LOH (Benn, 2021). In clinical practice, UPD can typically be ascertained using CMA testing (Del Gaudio et al., 2020). Computational algorithms can also be used to detect UPD greater than 10 Mb from trio exome or genome sequencing data (King et al., 2014; Magi et al., 2014; Bis et al., 2017; Del Gaudio et al., 2020). In the present study, both CMA and trio-WES were employed to identify UPD.

Among DGP that has 18 responsible genes, only two cases associated with UPD have been reported previously. A case of LGMD with a variant in *POMT2* and a case of a milder phenotype with a variant in *B3GALNT2* caused by UPD was reported in 2018 and 2022, respectively (Brun et al., 2018; D'Haenens et al., 2022).

In conclusion, to the best of our knowledge, we present the first case of MEB caused by UPD, providing valuable insights into the genetic mechanisms underlying this condition.

## Data Availability

The datasets for this article are not publicly available due to concerns regarding participant/patient anonymity. Requests to access the datasets should be directed to the corresponding author.
